# Modeling Two-Oscillator Circadian Systems Entrained by Two Environmental Cycles

**DOI:** 10.1371/journal.pone.0023895

**Published:** 2011-08-19

**Authors:** Gisele A. Oda, W. Otto Friesen

**Affiliations:** 1 Departamento de Fisiologia, Instituto de Biociências, Universidade de São Paulo, São Paulo, Brazil; 2 Department of Biology, University of Virginia, Charlottesville, Virginia, United States of America; Pennsylvania State University, United States of America

## Abstract

Several experimental studies have altered the phase relationship between photic and non-photic environmental, 24 h cycles (*zeitgebers*) in order to assess their role in the synchronization of circadian rhythms. To assist in the interpretation of the complex activity patterns that emerge from these “conflicting *zeitgeber*” protocols, we present computer simulations of coupled circadian oscillators forced by two independent *zeitgebers*. This circadian system configuration was first employed by Pittendrigh and Bruce (1959), to model their studies of the light and temperature entrainment of the eclosion oscillator in *Drosophila*. Whereas most of the recent experiments have restricted conflicting *zeitgeber* experiments to two experimental conditions, by comparing circadian oscillator phases under two distinct phase relationships between *zeitgebers* (usually 0 and 12 h), Pittendrigh and Bruce compared eclosion phase under 12 distinct phase relationships, spanning the 24 h interval. Our simulations using non-linear differential equations replicated complex non-linear phenomena, such as “phase jumps” and sudden switches in *zeitgeber* preferences, which had previously been difficult to interpret. Our simulations reveal that these phenomena generally arise when inter-oscillator coupling is high in relation to the *zeitgeber* strength. Manipulations in the structural symmetry of the model indicated that these results can be expected to apply to a wide range of system configurations. Finally, our studies recommend the use of the complete protocol employed by Pittendrigh and Bruce, because different system configurations can generate similar results when a “conflicting *zeitgeber* experiment” incorporates only two phase relationships between *zeitgebers*.

## Introduction

Synchronization of the physiology and behavior of organisms to the earth's periodic environment is achieved in part by the entrainment of circadian oscillators to species-specific combinations of daily photic and non-photic environmental cycles, known as “*zeitgebers*” [1]–[4].

The dominant *zeitgeber* that entrains circadian oscillators appears to be the light/dark cycle; however, the importance of food [5], [6] and temperature in circadian entrainment [7], [8] has received increasing recognition. In particular, temperature entrainment in the circadian organization of both ectotherms and endotherms [9]–[12], together with light effects, uncovers the necessity of considering the simultaneous action of two *zeitgebers*. The complexity of circadian systems comprising multiple oscillators entrained by two, or more *zeitgebers* can be understood through theoretical studies, where the contributions of *zeitgebers* and the internal circadian structure can be dissected. Such studies can also guide experiments and provide interpretations of the complex activity patterns of organisms.

Several experimental studies have altered the phase relationship between *zeitgebers* that occurs in nature, in order to assess their role in the entrainment of circadian rhythms [13]–[25]. The common experimental procedure is to artificially generate a phase difference of 12 h, in effect subjecting organisms to conflicting environmental time cues. In such “conflicting *zeitgeber”* experiments, it is commonly assumed that the circadian oscillator is phase-locked to the strongest *zeitgeber*. Intuitively, one might expect that some oscillators would follow the phase-displaced *zeitgeber*, whereas others would remain phase-locked to the unaltered *zeitgeber*.

**Figure 1:**
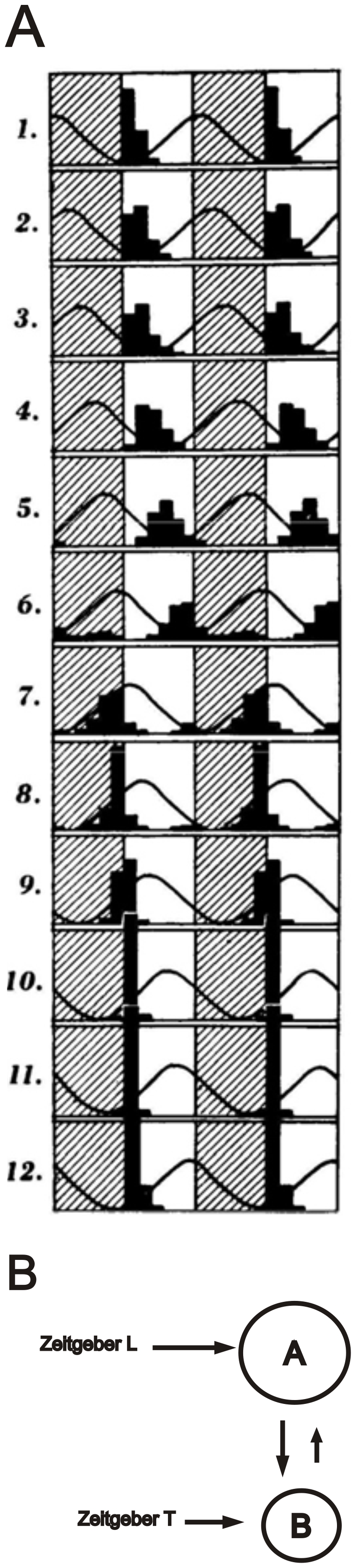
Experimental data and model of Pittendrigh and Bruce (1959). A) Eclosion rhythm of *Drosophila* populations under 24 h light/dark and temperature cycles. Each horizontal bar corresponds to two successive days. Light/dark cycles are shown by shaded areas and temperature cycles by a continuous curve, the latter being displaced by 2 h in each successive bar. Dark bars indicate the number of flies that eclosed within a 2 h time window. From Pittendrigh and Bruce (1959) with permission. B) Schematic diagram of two coupled A and B oscillators, entrained, respectively by *zeitgebers* L and T.

To the best of our knowledge, Pittendrigh and Bruce (1959) performed the most complete set of conflicting *zeitgeber* experiments, revealing complex dynamics in the phase of the overt rhythm. Their experiments were intended to assess the relative strengths of light/dark and temperature cycles in the entrainment of the circadian oscillators controlling adult eclosion in *Drosophila pseudoobscura.* Populations of flies were raised under 24 h light-dark and temperature cycles that are *zeitgebers* in this species [26], the latter being successively phase-shifted by 2 h steps relative to dawn (Fig. 1). The eclosion rhythm tracks the phase of temperature cycle during the first 6 conditions, but tracks the phase of light cycle in the subsequent 6 conditions. This observed switch in the phase association of eclosion rhythm, first to the temperature and then to the light cycle, has made the identification of the stronger *zeitgeber* for the control of eclosion in *Drosophila* uncertain until now.

In the present work, we describe our numerical simulations of limit-cycle oscillator models [27] that explain the main features of the complex behavioral results of Pittendrigh and Bruce (1959). More importantly, our simulations illuminate the spectrum of dynamics generated by conflicting *zeitgeber* experiments by revealing how coupled oscillators respond to progressive phase displacements between *zeitgebers*. Our simulations show how conflicting *zeitgeber* experiments disentangle the complex interactions between oscillators and *zeitgebers* if it is “complete”; that is, when the phase relationship between *zeitgebers* is progressively increased in small steps. We argue that a single phase displacement between two *zeitgebers* may generate misleading models of the circadian system.

## Methods

We performed numerical simulations inspired by the model of Pittendrigh et al. [28] and the experimental protocol of Pittendrigh and Bruce [13] with a system of coupled limit-cycle oscillators (A and B), with each oscillator affected by one *zeitgeber*, L or T (Figure 1B).

### Oscillator equations

The A and B oscillators were simulated by coupled Pittendrigh-Pavlidis equations (1–4), where *R* and *S* are state variables, and *a*, *b*, *c* and *d*, are parameters. 
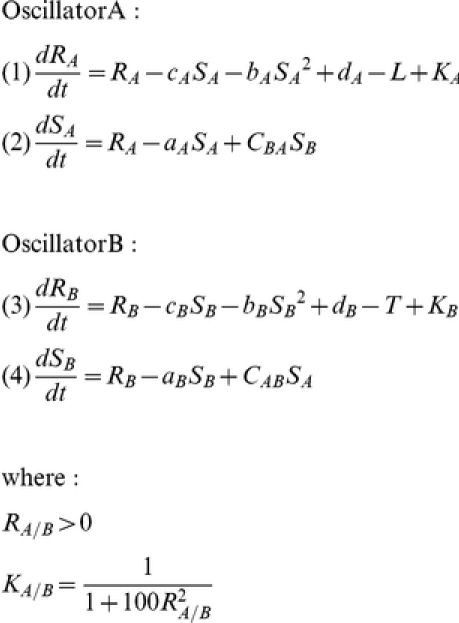
Parameters *C_AB_* and *C_BA_* set the coupling strengths of oscillator A to B and of oscillator B to A, respectively. Z*eitgebers* L, for oscillator A, and T, for oscillator B, are square-wave functions with a 24 h period. These equations differ from the Pavlidis equations [27] by a variable *K (Kyner)*, which is a small, nonlinear term that ensures numerical smoothness [29]. The R variables are explicitly constrained to be positive. These equations were developed originally for studies of the oscillator controlling the eclosion rhythm in *Drosophila* [27] and were employed in our modeling of the general properties of mammalian circadian oscillators [29]–[31].

As in former applications of this model, for the sake of simplicity and to better evaluate the effects of varying inter-oscillator coupling or *zeitgeber* strength, we assume that the oscillators are identical by fixing the parameters such that 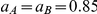, 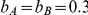, 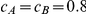 and 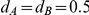. This parameter set generates an oscillator with intrinsic period ≈ 24 hr. Short, 1 h pulses (single pulse T-cycles with 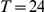 h [32]) were used in the simulations because they are the simplest mathematical models of daily *zeitgebers.* Z*eitgeber* amplitude (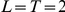) and coupling strengths (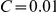 to 0.18) were chosen in a range that allowed a single z*eitgeber* to entrain the weakly coupled oscillators when alone. This choice of default values enabled exploration of coupling values that had the same effective magnitude as the z*eitgeber* strength.

**Figure 2:**
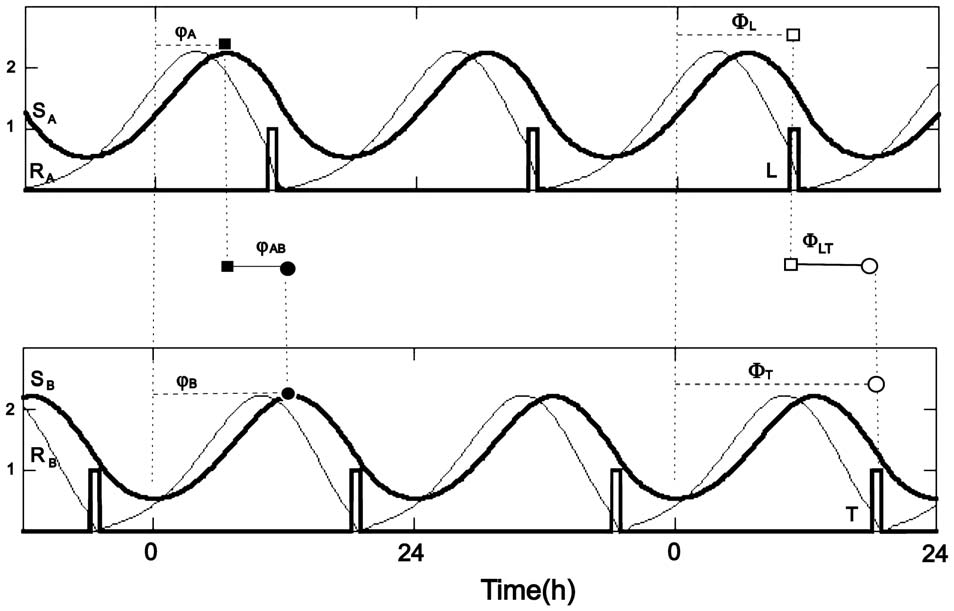
Schematic representation of the dynamics of oscillators and *zeitgebers*. Time course of oscillator A, *zeitgeber* L (upper panel), oscillator B and *zeitgeber* T (lower panel). *S* and *R* variables of each oscillator are represented, respectively, by heavy and light lines. *Zeitgebers* L and T are represented by rectangular pulses with periods  =  24 h. The phase of A, with respect to the acrophase (upper, filled squares) is given by *φ_A_*, that of B (lower, filled circles) is given by *φ_B_*_._ The phases of the *zeitgeber* pulses are indicated by *Φ_L_* (upper, open squares) and *Φ_T_* (lower, open circles). The phase difference between oscillators A and B is represented by *φ_AB_*, while the phase difference between *zeitgebers* L and T is represented by *Φ_LT_*.

Phases are defined with respect to the 24-hour day as follows (Fig. 2):

*φ_A_*  =  acrophase of oscillator variable S_A_, phase at which S_A_ takes its maximum value.

*φ_B_*  =  acrophase of oscillator variable S_B_.


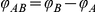
 = phase difference between coupled oscillators A and B.

*Φ_L_*  =  phase of *zeitgeber* L pulse onset.

*Φ_T_*  =  phase of *zeitgeber* T pulse onset.


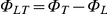
  =  phase difference between *zeitgebers* L and T. All phase values are given with respect to *Φ_L_*, which is assigned a value of 12 h. Thus, for example, a temperature onset phase of 8 h means that the temperature parameter *T* was set to 2, 4 h before light onset.

Simulations were performed with the *CircadianDynamix* software, which was developed to explore problems related to coupled and forced oscillators in chronobiology. It is an extension of *Neurodynamix II* [33], [34]. We used the Euler method for numerical integration, with 1000 integration steps per 24-hour day.

### Simulation Protocol

The phases of each oscillator (*φ_A_* and *φ_B_*) were evaluated under a series of entrainment conditions by successively increasing *Φ_T_*, in one-hour steps, from +12 to +24 h, and then from 0 to +12 h. The final state of each entrained condition was used as the initial state of the subsequent condition. The reverse sequence, from +24 to +12 h and from +12 h back to 0 h was also employed in order to test for dependence on initial conditions.

Furthermore, we focused on how *φ_A_* and *φ_B_*, at each *Φ_T_*, are affected by changes in the strength and symmetry of the inter-oscillator coupling and *zeitgeber* strength. The reference system was completely symmetrical, i.e. the oscillators, *zeitgeber*s and inter-oscillator coupling were identical; asymmetry was added by incremental changes in relative strengths of inter-oscillator couplings or *zeitgeber* amplitude.

## Results

### Symmetric System: identical oscillators, *zeitgebers* and coupling

To learn how the phase difference between *zeitgebers* (*Φ_LT_*) affects the phases of the A (*φ_A_*) and B (*φ_B_*) oscillators, as well as their phase relationship (*φ_AB_*), we set Pittendrigh-Pavlidis oscillator parameters to the simplest configuration: two identical oscillators with equal bidirectional coupling (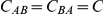). This system was subjected two identical, independent *zeitgebers*, whose relative phases were stepped from 0 to 24 h.

**Figure 3:**
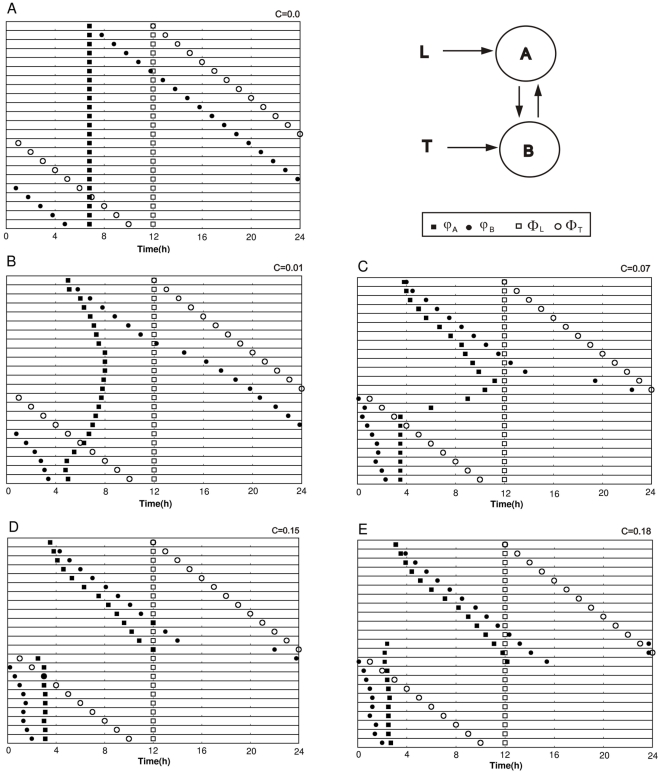
Symmetric limit-cycle system: Oscillator and *zeitgeber* reference phases at different coupling strengths. Steady-state oscillator phases (*φ_A_* and *φ_B_*) are represented by filled squares and circles, respectively; *zeitgeber* phases (*Φ_L_* and *Φ_T_*) are represented by open squares and circles, respectively (as in Fig. 2). In each of the 24 successive horizontal bars *Φ_T_* was increased by 1 h with respect to the phase of *zeitgeber* L, which was fixed at *Φ_L_* = 12 h. The duration and amplitude of both *zeitgebers* were fixed. Coupling strengths C were set to: A) 0.0; B) 0.01; C) 0.07; D) 0.15; and E) 0.18. Pittendrigh-Pavlidis model parameters here and in the remaining figures: *a* = 0.85, *b* = 0.3, *c* = 0.8, *d* = 0.5. *T* = *L* = 2.

We first investigated the effects of coupling strength between the two oscillators. The output of the symmetric system is shown in Figure 3, where the inter-oscillator coupling strength was increased from 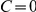 (panel A), 0.01 (panel B), 0.07 (panel C), 0.15 (panel D) and 0.18 (panel E). In the absence of coupling, each oscillator is phase-locked to its *zeitgeber* and the phase relationship between *zeitgebers* and oscillator acrophases remains constant (Fig. 3A). We found that even very weak coupling between oscillators was sufficient to modulate *φ_A_* and *φ_B_*, as revealed by the changing relationships between the *zeitgebers* and the oscillators (Fig. 3B, C). These effects become increasingly prominent with increasing inter-oscillator coupling strength (while the *zeitgeber* strength was maintained); eventually, the oscillators appear more strongly influenced by each other than by their *zeitgebers* (Fig. 3D, E).

**Figure 4:**
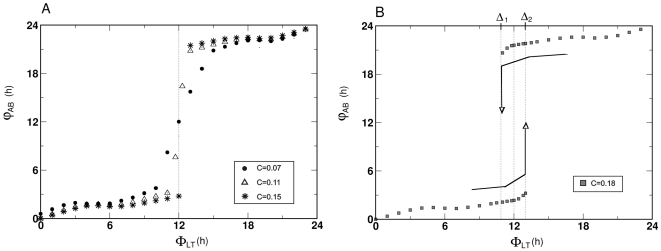
Symmetric Pittendrigh-Pavlidis system: Phase difference between oscillators (*φ_AB_*) as a function of the phase difference between *zeitgebers* (*Φ_LT_*). A) Increasing coupling strengths: filled circles,*C* = 0.07; triangles, C = 0.11; stars, *C* = 0.15. B) Strong coupling: *C* = 0.18. Δ_1_ and Δ_2_ indicate the interval where hysteresis occurs; arrows indicate the direction of the change in *Φ_LT_*. When *Φ_LT_* is below Δ_1_, phase jumps occurs at Δ_2_. When *Φ_LT_* is above Δ_2_ the converse path is taken and phase jumps occur at Δ_1_.This history dependence is the hallmark of hysteresis and gives rise to two φ_AB_ values (bistability) in the interval Δ_1_<*Φ_LT_*<Δ_2_. Pittendrigh-Pavlidis model parameters: *T* = *L* = 2.

A particularly interesting phenomenon is observed when *zeitgebers* T and L are near antiphase (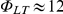
h). When inter-oscillator coupling is weak relative to the *zeitgeber* strength (Fig. 3A–C), oscillators attain large phase differences, up to 12 h, by non-linear, but smooth *φ_A_* and *φ_B_* changes. These phase differences are shown more clearly in Figure 4, where simulation data for the symmetric systems are replotted as *φ_AB_* as a function of *Φ_LT_*. These curves are sigmoidal, but with a sharp transition zone (for 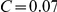 or greater). The maximum coupling value for which the antiphasic relationship between oscillators occurs (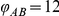
h when 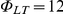
h) is 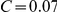 (Fig. 4A). Below this coupling value, φ_AB_ is a nearly linear function of *Φ_LT_*, but above it the oscillators become tightly phase-coupled, acting more as a single system. Under this tight coupling, large values of *φ_AB_* are not allowed (Fig. 3D and E, Fig. 4B).

Moderately abrupt changes in *φ_AB_* occur at 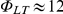
h, corresponding to the inflection point of the *φ_AB_* vs *Φ_LT_* curve, henceforth called “inflection phase”. In this vicinity, small changes in *Φ_LT_* result in relatively large changes in *φ_AB_*, hereafter described as a “phase jump”. The stronger the coupling, the steeper the phase jump, such as shown for 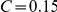.

For coupling values larger than 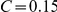, the results look rather different. In Figure 4B, (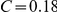, corresponding to data in Fig. 3E), as *Φ_LT_* increases from 0 to 24 h, *φ_AB_* remains near 0 h even beyond 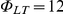
h, revealing an unexpected asymmetry in the graph. Then, as *Φ_LT_* increases further, at Δ_2_ (Fig. 4B) the value of *φ_AB_* jumps suddenly from just above 0 h to just below 24 h. With greater *Φ_LT_* values, *φ_AB_* remains below 24 h and a nearly linear function of *Φ_LT_*, attaining 0 h = 24 h for 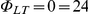
h.

A complete picture is revealed by considering the converse sequence of *zeitgeber* phase displacements, from 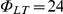
h to 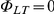
h. Although most *φ_AB_* values are identical to those obtained before, the phase jump occurs at 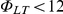
h (at Δ_1_, Fig. 4B). Thus *φ_AB_* attains two steady-states in the interval 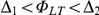, depending on whether the previous *Φ_LT_* was larger or smaller than 12 h. The complete graph, generated by increasing and decreasing *Φ_LT_* (indicated by arrows, Fig. 4B), restores the symmetry that was apparently missing; the picture now comprises bistability and hysteresis [35].

Bistability implies the existence of two stable steady states *φ_AB_*, for the same *Φ_LT_* values located in the “bistability zone” 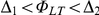, as is also seen in the explicit *φ_A_* and *φ_B_* values (lines 12,13 and 14 of Fig. 3E)_._ The phases attained by the system depend on initial conditions and this dependence on the path of parameter change is a hallmark of hysteresis (arrows, Fig. 4B). Preliminary exploration of alternative parameter sets of Pittendrigh-Pavlidis equations have shown, however, that the bistability zone is reduced as the free-running periods of oscillators deviate from 24 h.

### Asymmetric Systems

- Asymmetry in inter oscillator coupling strengths

We next examine the role of asymmetry by retaining equal *zeitgeber* strengths, but introducing asymmetry in the relative inter-oscillator coupling strengths. Using Pittendrigh-Pavlidis equations and departing from the symmetric case of a weakly coupled configuration 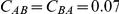, *C_BA_* was reduced from *C_AB_* to zero.

**Figure 5:**
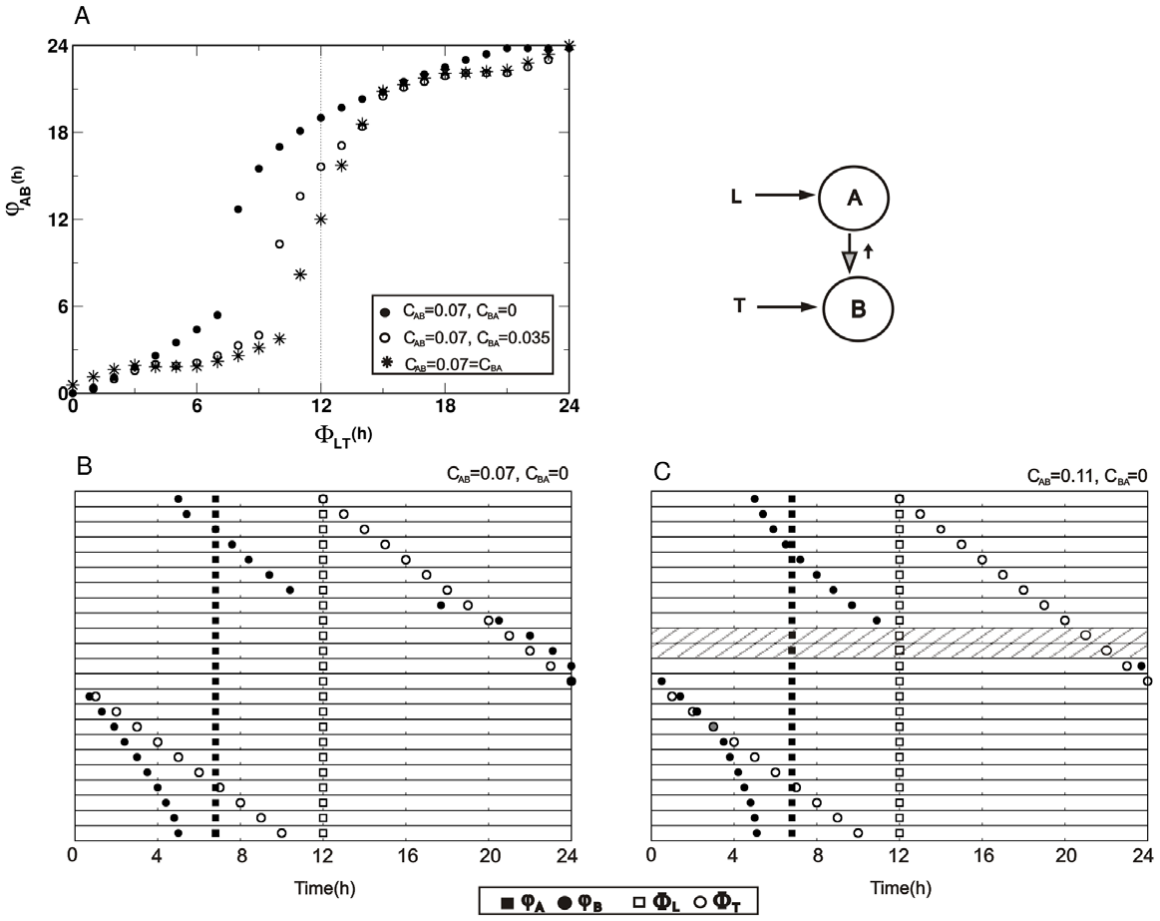
Asymmetrical inter-oscillator coupling in Pittendrigh-Pavlidis systems. A) Phase difference between oscillators (*φ_AB_*) as a function of the phase difference between *zeitgebers* (Δ*Φ_LT_*). *C_AB_* = 0.07 and C_BA_ varies as a fraction of *C_AB_* (see legend inside the figure). B) Oscillator and *zeitgeber* reference phases for a master-slave configuration, elicited by *C_AB_* = 0.07,*C_BA_* = 0. Oscillator phases (*φ_A_* and *φ_B_*) are represented by filled squares and circles, respectively; *zeitgeber* phases (*Φ_L_*and *Φ_T_*) are represented by open squares and circles, respectively. In each of the 24 successive horizontal bars *Φ_T_* was increased by 1 h with the phase of *zeitgeber* L fixed at *Φ_L_* =  12 h. The duration and amplitude of both *zeitgebers* were fixed. C) For a stronger value of unidirectional coupling *C_AB_* = 0.11 and *C_BA_* = 0, relative coordination (loss of stable entrainment) occurs in lines 9 and 10 (hatched). Pittendrigh-Pavlidis model parameters: *T* = *L* = 2.

When *φ_AB_* is plotted against *Φ_LT_* (Fig. 5A), the main qualitative feature of the symmetric system (Fig. 4) is recognized; namely, the sigmoidal shape of the curve. However, as asymmetry is increased by having 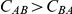, a progressive shift of the inflection phase occurs from 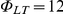
h to 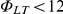
h (Fig. 5A). Maximum departure of the inflection phase from 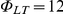
h occurs at the extreme, 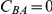 case, which corresponds to unidirectional coupling; that is, a “master-slave” configuration.

In the case of unidirectional coupling from A to B, *φ_A_* is always phase-locked to *zeitgeber* L, whereas *φ_B_* is modulated by both *zeitgeber* T and by L (via inputs from A; Fig. 5). Therefore, the dynamics of this asymmetrical system is now strongly dependent on the relative interaction strengths between *zeitgeber* T and *C_AB_*, which are pulling the slave B oscillator in opposite phase directions. Having fixed *zeitgeber* strengths (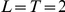), the master-slave system was now simulated for different coupling strengths *C_AB_* (Fig. 5B,C).

For a representative value of weak coupling (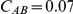), *φ_B_* is a smooth function of *Φ_T_* until 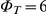
h, where a phase-jump occurs (7^th^ line, Fig.5B), and with the oscillator following the *zeitgeber* T smoothly thereafter. The sudden increase in *φ_B_* at 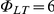
h, not at 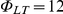
h, corresponds to the shift in the inflection phase of the sigmoidal curve.

The overall picture is similar for stronger coupling values (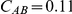, 0.15 and 0.18), except that T is not sufficiently strong to phase-lock *φ_B_* at some *Φ_T_* (9 h and 10 h, in this case). Thus, there is no stable entrainment at these *Φ_T_*, and oscillator B is in relative coordination [36] (Fig. 5C, lines 10 and 11, where φ_B_ was omitted). As coupling strength increases, the range of *Φ_LT_* that yields relative coordination enlarges.

- Asymmetry in zeitgeber strengths

**Figure 6:**
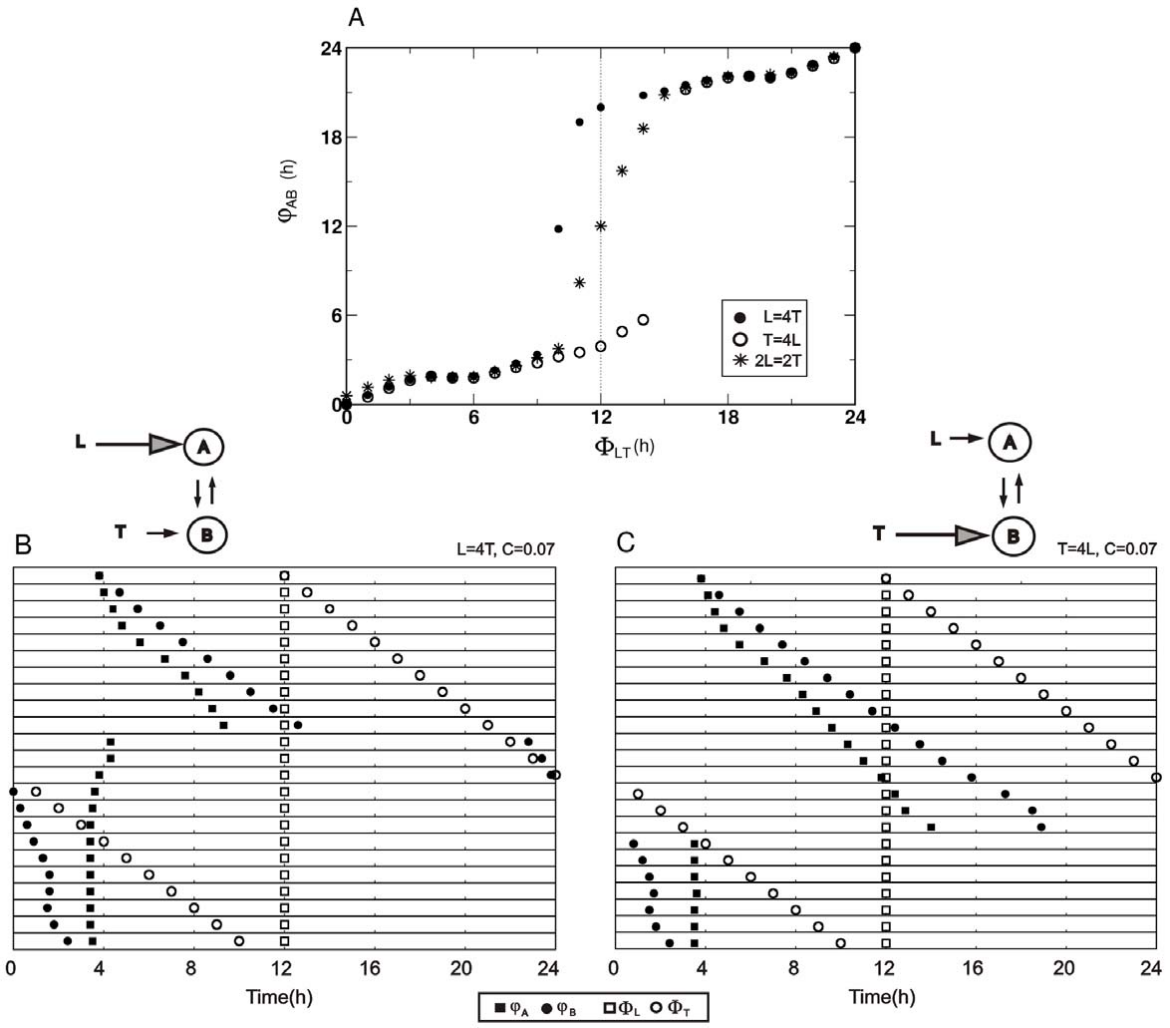
Asymmetrical *zeitgeber* strengths in Pittendrigh-Pavlidis systems. A) Phase difference between oscillators (*φ_AB_*) as a function of the phase difference between z*eitgebers* (*Φ_LT_*). Filled circles: asymmetric *zeitgeber* strengths *L* = 4,*T = *1; Open circles:*T* = 4, *L* = 1; Stars: symmetric *T* = *L* = 2. B) Oscillator and *zeitgeber* reference peak phases for an asymmetric *zeitgeber* configuration. Oscillator phases (*φ_A_* and *φ_B_*) are represented by filled squares and circles, respectively; *zeitgeber* phases (*Φ_L_* and *Φ_T_*) are represented by open squares and circles, respectively. In each of the 24 successive horizontal bars *Φ_T_* was increased by 1 h with the phase of *zeitgeber* L fixed at *Φ_L_* =  12 h. The durations of both *zeitgebers* were fixed while amplitudes were *L* = 4 and *T* = 1. C) *Zeitgeber* amplitudes *T* = 4 and *L* = 1. Pittendrigh-Pavlidis parameters: *C_AB_* = *C_BA_* = 0.07.

Asymmetry in *zeitgeber* strength, with symmetric inter-oscillator coupling, yields results similar to asymmetrically coupled oscillator systems; namely, a sigmoidal *φ_AB_* vs_._ *Φ_LT_* curve, with a shift in the phase of inflection. The inflection phase is greater for greater asymmetry between the two *zeitgeber* strengths (Fig. 6A). Phase jumps again occur at values that differ from 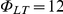
h. For comparison, oscillator phases are shown for a *zeitgeber* L which is 4 times stronger than T (Fig. 6B) and conversely, for a *zeitgeber* T which is 4 times stronger than L (Fig. 6C), resulting in a shift of inflection phase at *Φ_LT_* smaller or greater than 12 h, respectively.

### Interpreting Pittendrigh and Brucés (1959) *Drosophila* eclosion data

The following associations were made, in order to apply our model simulations to the *Drosophila* data.The phase relationship between *zeitgebers* was assigned the value 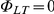 when the phase of temperature minima (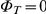) occurred at dawn (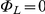), as represented in the first horizontal bar in Fig. 1A. Other complementary experiments described in [13] have shown that the phase of eclosion is determined by a temperature dependent oscillator B, which corresponds in our simulations to *φ_B_*. Furthermore, parallel simulations, not presented in this manuscript, have shown that the main dynamical features of periodic single-pulse *zeitgebers* are replicated by other cyclic wave forms, if appropriate amplitude adjustments are made.

We now focus on the following main features of the phase dynamics in Pittendrigh and Brucés (1959) data (Fig. 1A):

1. The phase shifts of the eclosion rhythm tracked the successive phase shifts of the temperature cycle in the 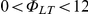
h interval (horizontal bars 1 to 6).
2. An abrupt phase jump of the eclosion peak was observed when *zeitgebers* attained maximum conflicting phase differences; i.e., near 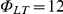
h (horizontal bar 7).
3. The eclosion phase remained nearly unaltered thereafter, independent of the phase of the temperature cycle along the 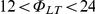
h interval (horizontal bars 8 to 12).

First, our simulations support the two-oscillator model of Pittendrigh and Bruce because an alternative model, comprising a single oscillator with temperature and light inputs is equivalent to a master-slave configuration system (Fig. 5) that does not replicate the above features.

Our simulations of the two-oscillator symmetric system with intermediate coupling relative to *zeitgeber* strength (Fig. 3D), qualitatively reproduces the findings in *Drosophila*. During the interval 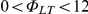
h, the phase-shifts of oscillator B track the phase shifts of the temperature *zeitgeber*. When 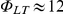
h, a “phase jump” of *φ_B_* occurs; thereupon *φ_B_* tracks the phase of the light *zeitgeber* for 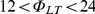
h. Our simulations have thus shown that the switch at 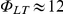
h of the preferential phase association of eclosion rhythm, first to the temperature and then to the light cycle can arise even in the most symmetric configuration, with identical oscillators coupled to identical *zeitgebers* (Fig. 3). By using a simple symmetric configuration, we show that additional complexity (unequal parameter values), even if it exists, is unnecessary for generating the observed complex phenomena. In other words, the results of the Pittendrigh and Bruce experiment do not indicate any *zeitgeber* dominance in *Drosophila*, neither of light/dark nor of temperature.

## Discussion

Our general study of conflicting *zeitgeber* experiments involving two differentially entrainable oscillators is applicable for a wide range of systems, such as: light/dark and temperature entrainable oscillators controlling eclosion rhythms in other insect species [24]; neuronal groups within the *Drosophila* brain, assessed by molecular concentrations [22], [25] or by emergent activity levels [23]; gene expression in the plant *Arabidopsis thaliana* [21]; dawn and dusk entrainable neuronal groups within the suprachiasmatic nucleus [37]; and finally, the light- and food- entrainable circadian oscillators in several species [5], [6], [15]–[20], [38], [39].

Most of the experimental studies that altered the natural phase relationship between photic and non-photic *zeitgebers* have restricted it to two *Φ_LT_* values, usually 0 and 12 h. Furthermore, they have also assumed linear relationships between the phases of *zeitgebers* and oscillators [18]–[23], [25], [37]. Such assumptions lead to the proposition that the phase of an output rhythm tracks the phase displacement of the stronger *zeitgeber* and thus two *Φ_LT_* conditions are sufficient for revealing the *zeitgeber* hierarchy. While this is correct when there is no coupling between oscillators (Fig. 3A), inter-oscillator coupling effects (Fig. 3B–E) add more complexity to the conflicting *zeitgeber* experiment, requiring a systematic change in *Φ_LT_* conditions to avoid ambiguous models.

The complex picture that emerged from Pittendrigh and Brucés experiment, comprising 12 *Φ_LT_* relations (Fig. 1A) is best replicated by the features of Fig. 3D, where light is as strong as temperature in the entrainment of the eclosion oscillator. However, if their experiment were restricted to only two phase relations between *zeitgebers*, for instance, 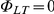 and 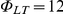
h (horizontal bars 1 and 7 of Fig. 1), the more limited set of phase results could be replicated by several other configurations.Furthermore, the problem persists if a different *Φ_LT_* pair is chosen for a conflicting *zeitgeber* experiment.

Some predictions and guidelines for a complete conflicting *zeitgeber* experiment arise from our simulations. The hypothesized general system is composed of two oscillators, differentially entrained by two *zeitgebers*. If the phase of the output rhythm (or of the oscillator itself) is plotted against the phase difference between *zeitgebers* Z_1_ and Z_2_, *Φ_Z1Z2_* a curve with different characteristics is expected.

- If the resulting graph is linear, instead of sigmoidal, there is no coupling between oscillators. Alternatively, the strength of one *zeitgeber* may not have been sufficiently strong to entrain its corresponding oscillator or the observed output is controlled by a master oscillator without feed-back from the unobserved slave (as it would occur in Fig. 5B, if the phase of *zeitgeber* L were shifted, instead of T).
- For a sigmoidal graph, the steeper the curve, with more pronounced phase jumps, the stronger is the inter-oscillator coupling with respect to the *zeitgeber* strengths (e.g., Fig. 3).Phase-jumps occurring at 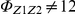
h are indicative of either an asymmetric system, with unequal coupling/*zeitgeber* strength between the oscillators, or a bistable system, with two potential entrained oscillator phases for an interval of *Φ_Z1Z2._*
- Dependence on initial conditions, with two possible entrained phases, is predicted to occur around the phase jump region if the inter-oscillator coupling is strong compared to the *zeitgebers*. This can be tested by pre-entraining with either of two *Φ_Z1Z2_*: in the natural phase relation or in *Φ_Z1Z2_* greater than the phase of inflection.
- Relative coordination is expected to occur for some *Φ_Z1Z2_* values, when coupling is strong with respect to *zeitgeber* strength.

Some other experiments have reported our predicted shift in the inflection phase, typical of asymmetric systems. This was observed by Bruce [14] and Pittendrigh [40], when they assayed, respectively, the circadian *phototaxis* rhythm in *Euglena viridis* and activity in cockroaches under light/dark and temperature cycles. Recently, Watari and Tanaka [24] verified that phase jumps occur at a 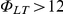
h in their conflicting *zeitgeber* experiments on the eclosion of onion fly *Delia antiqua*. Since only fixed initial conditions were used in these experiments, phase-jumps at 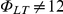
h could alternatively reflect a single branch of a bistable system (Fig. 3E), which would be testable by manipulating initial entrainment conditions.

There is much to be understood about the roles and interactions between non-photic and photic daily cues in the circadian organization of different species, in the context of different environments [15], [41]–[45]. Theoretical studies of multiple oscillator models explained phenomena that could not be accounted for by a single oscillator [32], [46]–[47] and widened opportunities for creative biological experiments [11], [12], [31], [48]–[51]. We are now facing the challenge of including multiple *zeitgebers* in this scenario, but in these early steps, our modeling study is appropriately limited to two general cases: symmetrical systems and those with simple asymmetries. Despite its simplicity, our system sufficed to give a glimpse of the rich dynamics behind multiple *zeitgeber* phenomena.
